# Functional specialization and generalization for grouping of stimuli based on colour and motion

**DOI:** 10.1016/j.neuroimage.2013.02.001

**Published:** 2013-06

**Authors:** Semir Zeki, Jonathan Stutters

**Affiliations:** Wellcome Laboratory of Neurobiology, University College London, London, WC1E 6BT, UK

**Keywords:** Parietal cortex, V4, V5, Cerebellum, fMRI, Grouping, Functional specialization

## Abstract

This study was undertaken to learn whether the principle of functional specialization that is evident at the level of the prestriate visual cortex extends to areas that are involved in grouping visual stimuli according to attribute, and specifically according to colour and motion. Subjects viewed, in an fMRI scanner, visual stimuli composed of moving dots, which could be either coloured or achromatic; in some stimuli the moving coloured dots were randomly distributed or moved in random directions; in others, some of the moving dots were grouped together according to colour or to direction of motion, with the number of groupings varying from 1 to 3. Increased activation was observed in area V4 in response to colour grouping and in V5 in response to motion grouping while both groupings led to activity in separate though contiguous compartments within the intraparietal cortex. The activity in all the above areas was parametrically related to the number of groupings, as was the prominent activity in Crus I of the cerebellum where the activity resulting from the two types of grouping overlapped. This suggests (a) that, the specialized visual areas of the prestriate cortex have functions beyond the processing of visual signals according to attribute, namely that of grouping signals according to colour (V4) or motion (V5); (b) that the functional separation evident in visual cortical areas devoted to motion and colour, respectively, is maintained at the level of parietal cortex, at least as far as grouping according to attribute is concerned; and (c) that, by contrast, this grouping-related functional segregation is not maintained at the level of the cerebellum.

## Highlights

► Subjects viewed stimuli containing groups defined by motion or colour. ► There was grouping related activity in cerebellum, in prestriate and parietal cortex. ► Activity in parietal cortex, but not cerebellum, was segregated according to modality.

## Introduction

In this study, we were especially interested to learn whether there is any specialization for grouping visual signals according to attribute. By grouping, we mean the process of assembling visual stimuli according to common elements. It is a general process that could be applied indifferently to any set of stimuli in any modality (Atkinson et al., 1976; Overvliet et al., 2012; ten Hoopen and Vos, 1979), and is therefore not necessarily tied to any particular visual or indeed sensory attribute. Its importance was emphasized by the Gestalt psychologists (Palmer, 2002; Wertheimer, 1950/1923) as a fundamental step in categorization.

In spite of the ubiquity and generality of the process, there was nevertheless the possibility that grouping of stimuli according to visual attribute may be a specialized process, neurologically separate from grouping according to other sensory inputs and possibly even dependent on the specific nature of the visual stimuli to be grouped. One cortical region that has been implicated in visual grouping in monkey is the parietal cortex (Freedman and Assad, 2006; Yokoi and Komatsu, 2009), and more specifically the intraparietal sulcus (IPS), which receives direct anatomical inputs from V4 and V5. The latter are among the most contrasting of visual areas in both monkey and human visual brains, the former being particularly active in the processing of signals related to colour, and the latter in signals related to motion (Bartels and Zeki, 2000; Brouwer and Heeger, 2009; Wade et al., 2008; Wandell and Winawer, 2011; Zeki, 1978b; Zeki et al., 1991). There is evidence to suggest that both may be involved in grouping in human and monkey (Castelo-Branco et al., 2002; Mirabella et al., 2007). Anatomical evidence shows that, in monkey, the two areas project to two contiguous and largely non-overlapping parts of the IPS (Shipp and Zeki, 1995). This suggests that in parietal cortex, too, operations related to colour and to motion may be segregated, with the implication that the specialization according to colour and motion that is evident in prestriate cortex may be maintained at the level of the IPS. If so, then this would extend the principle of functional specialization (Zeki, 1978a; Zeki et al., 1991) to parietal cortex and to grouping-related activity within it. Hence, our human study carried with it the dual promise, of revealing whether the two specialized visual areas are also involved in grouping visual signals according to their specializations, and whether this grouping involves activity in functionally specialized regions within parietal cortex. That, in turn, promised to give us insights into the broader question of whether, in executing a function that is formally general and not necessarily tied to a given attribute, the two specialized areas recruit the same or contiguous regions in the third areas to which they project, in this instance the parietal cortex.

## Material and methods

### Subjects

17 healthy volunteers, all with normal or corrected-to-normal vision and none with any neurological or psychological disorders were scanned. All subjects gave informed consent prior to participation and the Ethics Committee of University College London approved the study.

The first scanning session was used as a pilot experiment and data from it was not included in the analysis since it resulted in some changes to the stimuli presented in subsequent scanning sessions. An additional subject was rejected after pre-scan testing because he could only identify less than 60% of the test stimuli correctly. Of the 17 subjects who were scanned the mean age was 27 (minimum 20, maximum 54), 8 were male and none was left-handed. Subjects were tested with the Farnsworth–Munsell 100-hue colour discrimination test; their mean score was 33 (minimum 13, maximum 74).

### Stimuli

The stimuli consisted of 2 main categories: a) greyscale moving dots of varying levels of lightness in which there were between 0 (i.e. no grouping) and 3 groupings defined by common motion shared by all the dots in the groupings and b) coloured moving dots consisting of a field of randomly moving coloured dots of varying colours in which there were between 0 and 3 groups defined by a common colour shared by all the dots in all the groups. Additionally there were stimuli consisting of static colour and greyscale dots.

Stimuli were generated using Processing (www.processing.org) and consisted of 680 dots, each with a diameter of 10 pixels. The dots were initially positioned in a grid across a 1024 × 768 pixel area and their locations adjusted by a random amount of between − 10 and 10 pixels on both the x and y-axes. This resulted in a field of dots with no clear structure but with more even coverage than would be produced by full randomisation of dot positions. In the coloured condition, each dot was assigned a random colour from the full 8-bit red, green, and blue (RGB) range while in the greyscale condition each dot was assigned a grey level equal to the lightness portion of the CIE 1976 (L*, a*, b*) colour space value of a randomly selected colour from the coloured condition stimuli. After the dot fields were created, between 0 and 3 circular regions with a diameter of 100 pixels were defined to serve as the groupings. The positions of these regions were random but with the constraint that no two regions should be coincident and no region should extend beyond the edge of the visible area. In the colour grouping condition the regions remained static though the dots within them moved while in the motion grouping condition they were only used during initialisation to define the groups (see below). Each dot's direction was adjusted by a random amount of between − 0.2 and 0.2 rad once per frame. The direction of motion was weighted by adding a vector along the x-axis of 1.5 pixels per frame to each dot's velocity vector so that the dots tended to move from left to right. After randomisation of the direction and applying the left-right weighting the velocity was normalised such that every dot was moved by 1.7 pixels per frame. In all conditions the stimulus was displayed for 5 s followed by a 3 s blank.

We emphasize that the dots were always in motion in all stimuli in which there was grouping according to colour or to motion. The sole differences was that in motion grouping stimuli some dots within the array of dots were grouped together in such a way that all the dots within that group moved in the same direction. In the colour grouping stimuli all the dots were in motion but some of the dots were grouped together according to colour.

In the moving greyscale condition, dots that were initialised within a grouped region were given a different movement characteristic, thus: all dots marked as within a grouping region at the start of that stimulus followed the same random motion. Where there were 2 or 3 groupings present, all dots within the groupings moved in the same direction. No left to right weighting was applied to the motion direction of the grouped dots to make it possible to distinguish them from the other randomly moving non-grouped dots. At the beginning of a greyscale stimulus the dots were initialised by advancing them through 100 frames of motion to ensure that no ‘holes’ were left in the overall distribution of the dots as a result of the dots that moved in a group (this was not necessary in the colour condition as all dots had the same motion characteristics). This 100 frame period was not conducted in real time or displayed to the subject. In cases where the dots moved out of the visible area they were wrapped around to the opposite edge. An exception to this was the moving greyscale condition in which the dots within groups were prevented from moving off the edge of the screen by reversing the group's direction when an edge was encountered.

In the colour grouping condition dots which moved into one of the grouping regions changed colour to a single randomly selected colour (selected from the full RGB space) which characterised all dots within that grouping. When dots left the grouping regions they reverted to their previously assigned colour. When more than one grouping was present the same colour was used for all the groups. The resulting effect was of 1 to 3 coloured patches made up of dots in a field of randomly coloured dots.

The positions and colours of the dots were exported to a file that could be read by a MATLAB script for playback to the subject.

Three additional categories of stimuli were used: static coloured dots, static greyscale dots and blank. The static stimuli were created by randomly selecting a frame from either the greyscale or coloured stimuli in which there were no groups defined; there were therefore no groups in the static conditions.

A video demonstrating the colour and motion stimuli is available from http://www.vislab.ucl.ac.uk/grouping_movies/grouping.html.

### Stimulus presentation

Stimuli were presented using Cogent 2000 (http://www.vislab.ucl.ac.uk/cogent_2000) running in MATLAB (Mathworks, Natick, MA, USA). In the scanner the stimuli were back-projected onto a screen using an LCD projector (at a resolution of 1400 × 1050 pixels) that subjects viewed through an angled mirror. The display arrangement gave a visual field of 24 × 19°, resulting in a single dot having a diameter of approximately 0.2° and moving at a speed of 1.7°sec^− 1^.

Immediately prior to scanning subjects were instructed to indicate how many groups they were able to detect using a keypad, and to keep their eyes fixated on the central cross. The stimulus presentation began with the entire display black while the scanner acquired seven volumes of data, which were discarded to remove the unwanted effects of T1 equilibration. The stimuli were displayed in a semi-random order generated by an algorithm that favoured short runs of the same stimulus category. A white fixation cross (150 pixels across, vertically oriented) appeared in the centre of the screen when a stimulus was present (including in the blank condition). The stimuli were presented against a black background. The screen was black between stimuli. During each stimulus presentation subjects used the button box to indicate whether they saw zero, one, two or three groups, by pressing one of four buttons.

### Tracking of eye movements

Eye movement data was collected using an EyeLink 1000 system from SR Research (http://http://www.sr-research.com/) equipped with an MR compatible camera. The eye position was measured at 500 Hz and recorded using the Spike2 software (http://www.ced.co.uk). Tracking data was collected for all 32 scanning sessions (16 subjects and 2 scanning session per subject).

### Retinotopic mapping

We undertook retinotopic mapping mainly to determine whether there was grouping-related activity in areas V2 and V3. We are aware of the difficulties experienced by many in mapping V4, V5 and the subdivisions of the intraparietal cortex retinotopically. Fortunately this was not necessary in our case, because it was sufficient to use localizer contrasts (static colour dots versus greyscale dots for V4 and moving greyscale dots versus static greyscale dots for V5) to get the positions of the V4 complex (V4 + V4a, Bartels and Zeki, 2000) and the V5 complex (corresponding to V5 plus its satellite regions (Kolster et al., 2010)) and show that colour-related activity was in the territory of the former and motion-related activity in that of the latter. Moreover, grouping-related activity in the intraparietal sulcus invaded the territory of all recognized subdivisions there (Swisher et al., 2007), making it un-necessary to isolate individual subdivisions (see results (Results section)). The mapping stimulus used and the analysis process are described in the supplementary data Section 1.

### Scanning details

MRI data was acquired using a 3 T Siemens Magnetom Trio scanner (Siemens, Erlangen, Germany) fitted with a 32-channel head-coil. Prior to scanning, a localizer sequence was performed to locate the subject within the scanner and the magnetic field was mapped to correct for the effect of the subject on the static field in the scanner. An echo-planar imaging (EPI) sequence was used for the functional imaging (echo time TR = 68 ms, TE = 30 ms, volume time = 3.264 s) using 48. Voxel resolution was 3 mm × 3 mm with a 2 mm slice thickness and 1 mm inter-slice gap. Heart rate, breathing and eye movement were recorded during the functional scan. Two sessions of functional data were collected with a short break between the sessions. A different set of stimuli was used in each session and a new order was generated but they were otherwise identical. A T1-weighted anatomical image was acquired for each subject after the functional scanning was completed (176 slices, 1 × 1 × 1 mm resolution, TE = 2.48 ms, TR = 7.92 ms). After the structural image was acquired, two runs of retinotopic mapping data were acquired using the same EPI sequence as was used to acquire the main functional data. Retinotopic mapping data was only collected for 8 of the subjects (see also the discussion in Retinotopic mapping section).

### Analysis

The eye tracking data was analysed in MATLAB using the sigTOOL software (Lidierth, 2009).

The MRI data was prepared for analysis in SPM8 (http://www.fil.ion.ucl.ac.uk/spm/software/spm8/). Stimulus events were modelled as boxcar functions. The stimuli were separated into 6 main contrast vectors — moving colour with grouping, moving colour without grouping, moving greyscale with grouping, moving greyscale without grouping, static colour and static greyscale. The subjects' keypresses and head movement parameters (from the realignment pre-processing step) were included in the General Linear Model (GLM) as regressors of no interest. As the event duration was short, time derivatives were added to the GLM for each contrast vector. A high pass filter with a cutoff of 120 s was applied to the data to control for slow signal drift.

An additional GLM was constructed in which the stimuli were divided into 4 main contrast vectors — moving colour, moving greyscale, static colour and static greyscale. For the two conditions with movement, the number of groups (from zero to three) was added as a linear parametric modulator. Regressors of no interest, high pass filtering and time derivatives were added to this GLM as described above.

Maximum likelihood estimates of the parameters were then taken to the second (between subject) level for random effects inference using the summary statistics approach. This involved taking contrasts or mixtures of parameter estimates summarizing condition-specific effects in each subject and creating SPMs of unpaired t-statistics.

All co-ordinates are given in Montreal Neurological Institute (MNI) space.

### Conjunction between colour grouping and motion grouping

The contrasts for colour grouping > no colour grouping and motion grouping > no motion grouping, as well as the linear parametric activity related to number of groupings for both colour and motion, were assessed for conjunctions (Price and Friston, 1997) at the single-subject level by masking the colour grouping contrast with the active voxels from the motion grouping contrast (using an uncorrected mask p-value of 0.05).

## Results

### Task difficulty

The reaction times for accurate responses did not differ significantly by number of groupings (1 group: 2926 ms, 2 groups: 2923 ms, 3 groups: 2790 ms), from which we conclude that the stimuli with different groupings did not differ in task difficulty.

The mean accuracy in terms of responses did not significantly differ for stimuli with 1 grouping (80%) and those with 3 groupings (85%) but was significantly higher for stimuli with two groupings (89%) than those with one (p = 0.011) or three groupings (p = 0.048). This ordering of accuracy in terms of number of groupings bore no relationship with the strength of activations relating number of groupings to the BOLD signal. This reinforces the conclusion that the results cannot be accounted for in terms of task difficulty.

### Eye tracking

For the 14 sessions where good eye-tracking data (defined as > 80% valid; invalid data occurred as a result of the subject blinking or the tracker otherwise not detecting the pupil or corneal reflection) was collected, the mean correlation coefficient between the standard deviation of the eye position and a binary signal indicating the presence of grouping was − 0.08 (std. dev. 0.16), indicating that the subjects did not significantly increase or decrease their eye motion in response to the presence of groupings or their numerosity.

### Retinotopic mapping

**Fig. 1:**
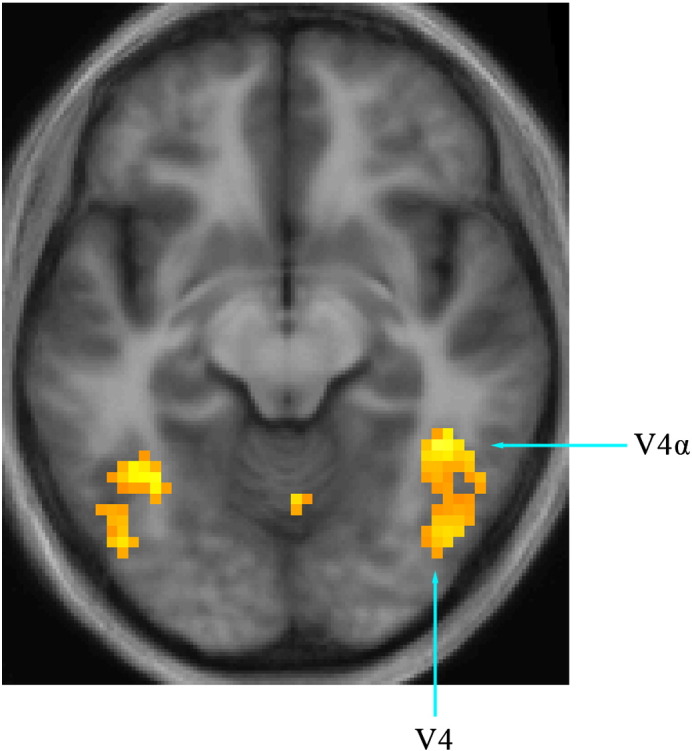
Activity produced in the fusiform gyrus (V4 complex) in the contrast colour grouping > no grouping. Slice at 45, − 49, and − 11. Threshold p < 0.001 uncorrected. The coordinates of the posterior activation correspond to V4 (this study: (45, − 67, − 14); Bartels and Zeki, 2000: (28, − 51, − 22)) and those of the anterior one to those of V4α (this study: (42, − 46, − 11); Bartels and Zeki, 2000: (34, –75, − 21)).

We were able to identify V1 and V2 in 6 of the 8 subjects used for retinotopic mapping, V3 in 4 subjects and V4 in two. Of the two subjects in whom it was possible to map V4, neither showed any activity in their SPM contrasts when a masking for V4 was applied. This is almost certainly due to the fact that in these two subjects the hottest activity within the V4 complex fell in V4α, which is poorly organized retinotopically (Bartels and Zeki, 2000) (Figs. 1 and 3a[ii]).

### fMRI results

We report cluster activations which were significant at p < 0.05 for family-wise error rate (FWE), with a Bonferroni correction for multiple comparisons. Tables 1a, 1b, and 1c summarize the activation produced by each contrast.

We were mainly interested in the following contrasts:

#### Localizer contrasts

**Fig. 2:**
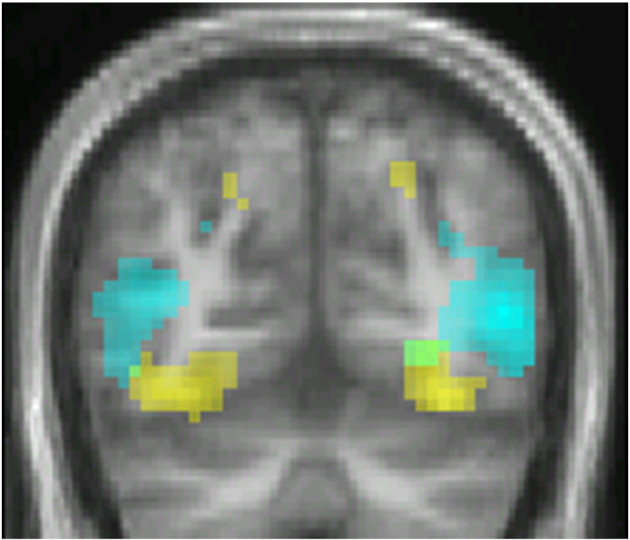
Coronal section at 62 mm MNI space showing activation for colour > greyscale in V4 (yellow) and greyscale motion > static in V5 (cyan). Threshold at p < 0.001 uncorrected.

Colour > greyscale static and (b) motion > greyscale static, which acted as localizers for visual areas V4 and V5. The former led to activation within dorsal and ventral V2 and in V4 while the latter led to activation within V2, V3, and V5 and in medial parietal cortex (Fig. 2 and Table 1a).

Although we were able to define V4 retinotopically in two subjects, retinotopic identification was more difficult for V5 (see Discussion section). But the activations produced by colour and motion in this study correspond to previously reported co-ordinates for V4 and V5 (Bartels and Zeki, 2000; Brouwer and Heeger, 2009; Kolster et al., 2010; McKeefry and Zeki, 1997; Moutoussis and Zeki, 2008; Tanabe et al., 2011; Wade et al., 2008; Watson et al., 1993).

#### Grouping contrasts

**Fig. 3:**
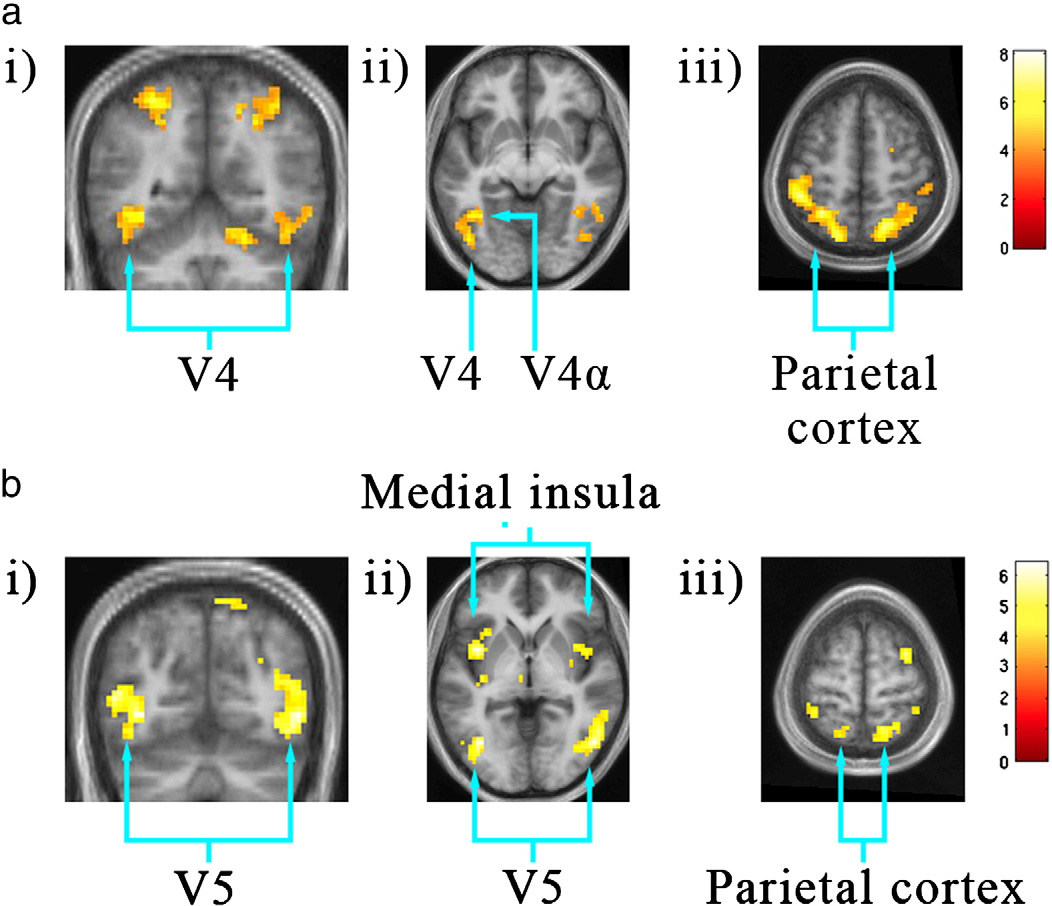
a: Averaged activity produced by the contrast colour grouping > no grouping. Threshold p < 0.001 uncorrected. i) Coronal section at − 55 mm MNI. ii) Horizontal section at − 8 mm MNI. iii) Horizontal section at 54 mm MNI. b: Averaged activity produced by the contrast motion grouping > no grouping. Threshold p < 0.001 uncorrected. i) Coronal section at − 67 mm MNI. ii) Horizontal section at − 2 mm MNI. iii) Horizontal section at 64 mm MNI.

The contrast colour grouping > no grouping led to activity in (a) V4 bilaterally (Fig. 3a[i–ii]); (b) prominent activity oriented antero-posteriorly within parietal cortex bilaterally, along the banks of the IPS and extending onto the superior parietal lobule (Figs. 3a[iii] and 4). Although there were hot spots along the spread of activity in the IPS, it was distributed over all four of the recognized subdivisions of the IPS (Swisher et al., 2007) (see Table 2 and also Discussion section); (c) in the right inferior frontal gyrus and Crus 1 of the cerebellum (Figs. 3a[i], 4 and Table 1b).

The contrast motion grouping > no motion grouping led to activation within (a) V5, bilaterally (Fig. 3b[i–ii]); (b) parietal cortex, distributed antero-posteriorly along the IPS as with the activity produced by the contrast colour grouping > no grouping, but less prominently; and (c) in medial insula bilaterally (Figs. 3b[ii], 4 and Table 1b).

#### Deactivations

The contrast no colour grouping > grouping led to bilateral deactivation in V1 (Table 1b). The contrast no motion grouping > grouping led to a deactivation of V1 in the left hemisphere (Table 1b).

#### Parietal activation

It was not possible to map any of the parietal cortex areas using the retinotopic mapping data, possibly because of ‘drop-outs’ in the maps and substantial variability between individuals (Swisher et al., 2007) (see also Discussion section). But the antero-posterior distribution of activity, orthogonal to the axes of the areas in IPS, the extension of activity onto the superior parietal lobule, and the close correspondence between the co-ordinates obtained here and those given by Swisher et al. (2007) for the four IPS areas (IPS1–IPS 4) shows that there was activity in all four (see Figs. 3a[iii], 4 and Table 2).

#### Conjunction of motion grouping and colour grouping in parietal cortex

**Fig. 5:**
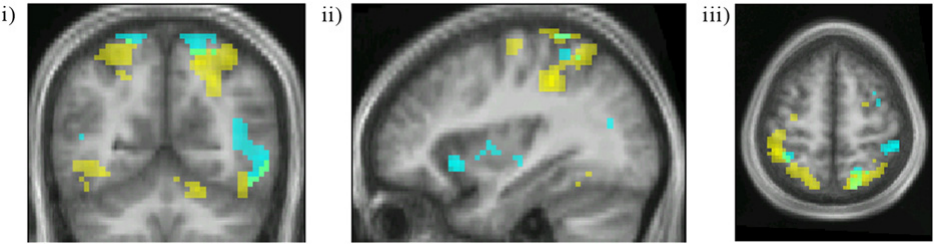
Overlaid contrasts showing conjunction of activity produced by the contrasts colour grouping > no grouping (yellow) and motion grouping > no grouping (cyan). Conjunction of activity in IPS shown is in iii. Contrasts thresholded at p < 0.001 uncorrected. Slices in MNI co-ordinates at − 58 (coronal, i), − 33 (parasaggital, ii) and 57 (horizontal iii).

Only 1 out of 16 subjects showed any significant activation in the masked contrast (at − 66 − 25 37; − 48 − 34 49; and 69–16 31 MNI space) (Fig. 5), suggesting that the activity in parietal cortex resulting from colour and motion grouping occurs in neighbouring but not overlapping, or in only partially overlapping, regions there.

#### Parametric relationship between BOLD signal and number of groupings

**Fig. 6:**
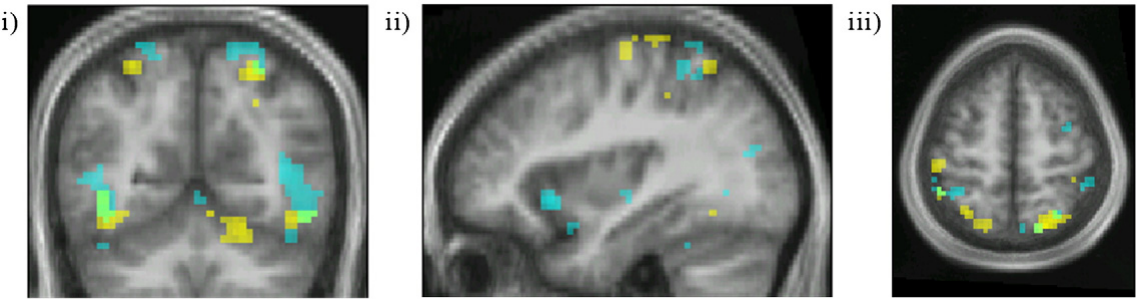
Overlaid contrasts showing conjunction of the contrasts showing voxels with activity linearly related to the number of colour groups (yellow) and the number of motion groups (cyan). Contrasts thresholded at p < 0.001 uncorrected. Same slices as in Fig. 5.

**Fig. 7:**
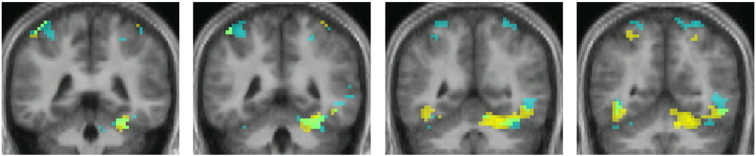
Overlaid coronal sections through the cerebellum showing the conjunction of activity for number of colour groups (yellow) and number of motion groups (cyan). Contrasts thresholded at p < 0.001 uncorrected. Slices at 42, 45, 51, and 55 mm MNI.

We also wanted to learn (e) whether there was any relationship in the active areas between the BOLD signal and the number of groupings detected. Figs. 5 and 6 show the conjunction of areas in which strength of activity was parametrically related to the number of groupings, for both colour and motion (plots showing the strongest and weakest relationships between BOLD response and number of groupings can be found in supplementary data Section 2). They show that activity related to colour and to motion groupings is segregated within prestriate cortex, in V4 for colour groupings and in V5 for motion groupings. They are maintained largely segregated within parietal cortex as well (see also Table 1c). This is in contrast to the parametrically related activation produced in the cerebellum by the same groupings, which largely overlap (Fig. 7).

We did not detect any activity that was parametrically related to grouping in areas V2 and V3.

## Discussion

The grouping of elements in a visual scene having a common property, to distinguish or segment them from other elements of the same scene not having that common property, is a critical process for visual recognition. Given the functional specialization of the visual brain (Zeki, 1978a; Zeki et al., 1991) and especially the specialization for colour and motion, it was interesting to learn whether grouping, being a process that can be applied to any of a variety of visual signals, is also subject to specialization. It is, in theory, entirely plausible that grouping may be an activity that is undertaken outside the specialized visual areas and indeed outside the visual cortex itself, for visual attributes are not the only ones that are grouped together. But a more intuitive supposition is that areas specialized for a given attribute, in our instance for colour and visual motion, would be involved in the grouping, either exclusively or with the cooperation of other areas, visual or otherwise. The results we obtained show that the latter was indeed the case. Before discussing these results, we discuss possible confounds.

### Attention

Determining the number of groupings naturally demands attention, making it interesting to consider the role that attention per se may have played in the pattern of activity that we observed. Attentional load enhances strength of activity in both V4 (Bartels and Zeki, 2000, 2005; Schoenfeld et al., 2007) and V5 (Bartels and Zeki, 2005; Chawla et al., 1999; O'Craven et al., 1997) but this enhancement is always accompanied by activity in frontal cortex, principally in the frontal eye fields (FEF), which is part of the attentional system (Buchel et al., 1998; Kastner and Ungerleider, 2000; Kastner et al., 1999; Saygin and Sereno, 2008; Zanto et al., 2011). We did not observe any activity there. This would seem to rule out a generalized top-down attentional effect which the FEF apparently exerts, the attentional mechanisms of parietal cortex being stimulus driven (Ruff et al., 2008). It is of course difficult to isolate attention from determining number of groups, but the fact that the attentional requirements were equalized across conditions in our paradigm, added to the fact that parietal activations induced by grouping according to motion or to colour occupied non-overlapping territories there, make it unlikely that the results we observed are due to general attention towards spatial location or to general top-down attentional influences from the frontal cortex. We discuss the possible attentional component in the cerebellar activation below.

The brain's attentional system has been divided into two components, both of which involve parietal cortex but which differ with respect to the part of frontal cortex that each involves (Corbetta and Shulman, 2002): the dorsal attentional system, thought to have a generalised attentional function, involves the FEF, while the ventral one, thought to be important for detecting behaviourally relevant stimuli, involves the inferior frontal gyrus. It is only part of the latter, ventral, attentional system that was active in our study, but only with colour grouping, not the motion one. Activity for the latter was only elicitable with the use of a 12 mm small volume correction, and even then it was only trend significant. A similar result has been reported by Zanto and Gazzaley (2009).

In summary, it would seem that our results are not explicable by a general attentional mechanism, though a part of ventral attentional system was active presumably because of the behaviourally relevant paradigm in our study. Hence, even accepting that a part of the attentional system is (necessarily) engaged in a task that demands attending to groupings, the fact still remains that, within parietal cortex, the attentional component engages different parts of it.

### Coherence

The grouping of signals according to direction of motion or to colour naturally involves some degree of coherence. The relationship between strength of activity in V5 and coherence is problematic; some have reported that the strength of activity in it is related to the degree of coherence (Rees et al., 2000) and others that coherent patterns of dot motion are less potent in activating V5 than incoherent ones (McKeefry et al., 1997; Smith et al., 2006). While this makes it difficult to argue for or against coherence as a determining factor in our results, we note that, in the colour grouping condition, the coherence in the motion pattern was the same regardless of whether there was a grouping according to colour or not, and regardless of the number of groupings, and yet the parametric activity related to groupings was in V4, and within a segregated zone of IPS. This would seem to argue against an explanation of our results based on motion coherence. Moreover, those who have reported a positive relationship between degree of motion coherence and strength of activity in V5 have also shown that, as a corollary, there is a de-activation in anterior cingulate cortex (Rees et al., 2000), which we did not observe.

### Extent of visual field stimulated

An increase in the number of groupings entails an extension in the area of the visual field stimulated by groupings. We note, however, that our groupings (whether of colour or of motion) were embedded within a larger stimulus that included the same coloured dots which, though in motion, were not grouped together. Hence the total extent of the visual field stimulated was the same regardless of whether there was a grouping or not. We nevertheless acknowledge that, within the equal extent of visual field stimulated, three groups will involve a larger extent of the visual field stimulated by coherence than one group or no groups. Acknowledging this makes little difference to the principal conclusion of this study, namely that grouping or coherence according to colour or motion engages different compartments within the intraparietal sulcus and the same subdivision of the cerebellum.

### Retinotopic mapping

The parietal cortex has several subdivisions (Silver and Kastner, 2009; Silver et al., 2005; Swisher et al., 2007). We tried, through retinotopic mapping, to learn whether the activity was distributed in all or concentrated within some of them. This was no easy task. Indeed in the study by Swisher et al. (2007), only 5 of the 20 subjects studied had “relatively” clear parietal maps. Although we could not distinguish the subdivisions of parietal cortex [see Material and methods section] comparison of our results with those of Swisher et al. (2007) shows that all four IPS areas were activated by the grouping task, and that activity in the three anterior ones was parametrically related to the number of groupings (see Table 2).

### Grouping activity in areas V4 and V5

Numerous studies have now shown that colour stimuli activate V4 and motion stimuli activate V5 (Brouwer and Heeger, 2009; Kolster et al., 2010; McKeefry and Zeki, 1997; Moutoussis and Zeki, 2008; Wade et al., 2008; Watson et al., 1993), and hence that there is a functional specialization within visual cortex for processing these two different attributes of vision (Zeki, 1978b; Zeki et al., 1991). Even in spite of a report showing that, in monkey, only cells of the lateral intraparietal sulcus (LIP) and not those of V5 are involved in motion categorization (Freedman and Assad, 2006) it would have been surprising if the two specialized visual areas (V4 and V5) were not also involved in grouping according to their specialization, for grouping must depend upon prior processing according to specialization. Indeed, previous physiological and imaging studies have shown that the activity of cells in V4 can be related to the selection of targets for behaviour (Mirabella et al., 2007) and that of cells in V5 to grouping (Castelo-Branco et al., 2002). The grouping-related activity for colour in V4 and motion in V5 may in fact depend, as well, upon an exchange of signals between these areas and areas within the IPS, with which they are reciprocally connected (Shipp and Zeki, 1995), the nature of which will no doubt be decided by future studies employing techniques with a higher temporal resolution. The results here, as well as parallel ones on concept formation based on colour and motion, where subjects were required to recognize motion or colour stimuli according to colour or motion concepts which they had formed (Cheadle and Zeki, submitted for publication), show that there may be a neural difference in the two related processes of grouping and concept formation, even if the latter is based on the former. For our results on concept formation, while showing an involvement of the more anterior parietal areas (IPS4) in concept formation based on colour and motion, do not show a significant involvement of V4 and V5 in concept formation based on the attributes of colour and motion.

### Grouping activity in parietal cortex

**Fig. 8:**
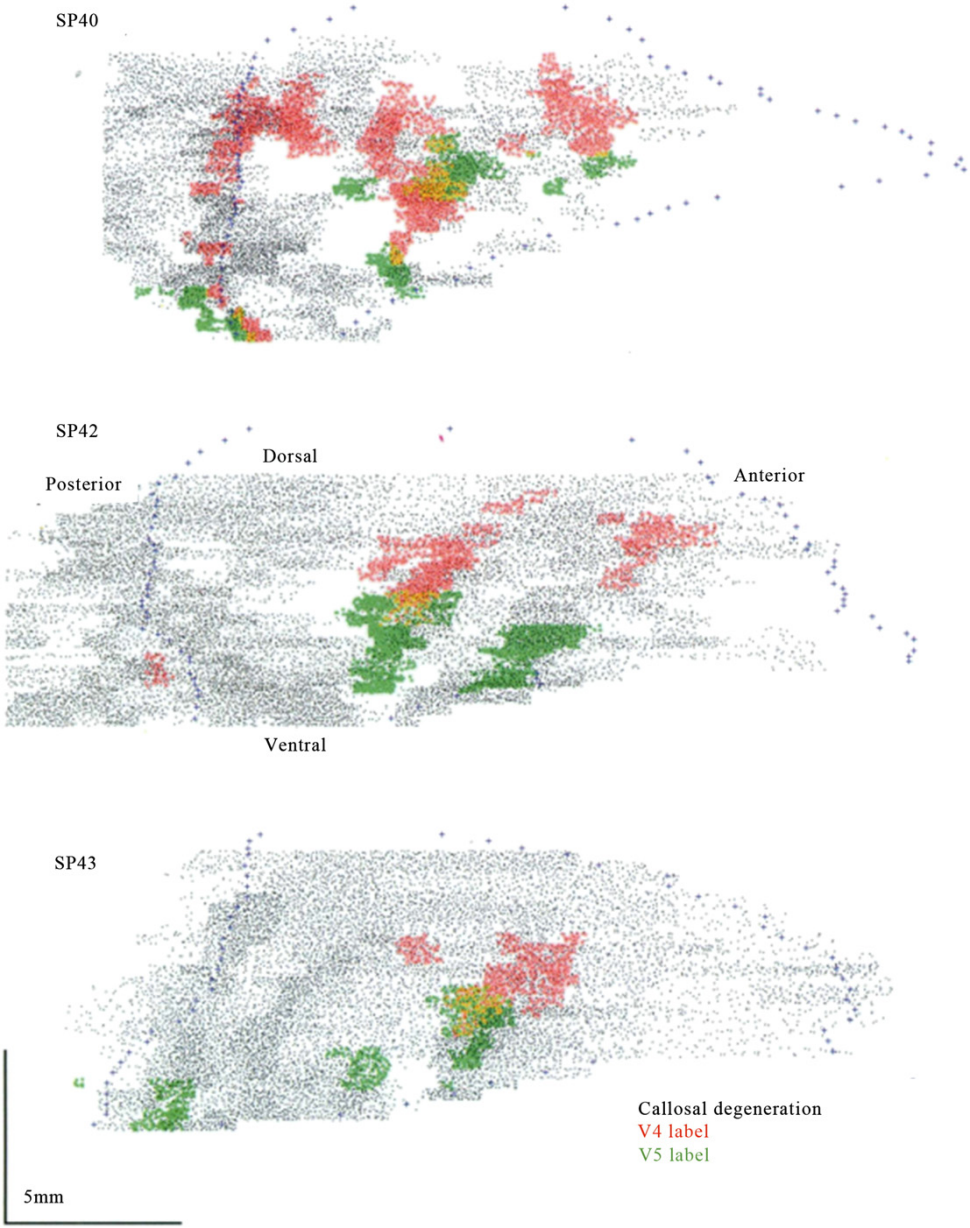
Diagrammatic, flattened representation of the projections to monkey intraparietal cortex from V4 and V5, revealed by injecting the two areas with the horseradish peroxidase tracer. From Shipp and Zeki, 1995.

Parietal cortex has been traditionally associated with the ‘where’ system although many recent studies have shown that it has functions that do not sit neatly within this classification (Fattori et al., 2012; Konen and Kastner, 2008). Among these is grouping (Freedman and Assad, 2006; Yokoi and Komatsu, 2009). Our present study shows that it is involved in the grouping of visual signals based on both colour and motion. This involvement is no doubt aided by the fact that both V4 and V5 connect reciprocally with parietal cortex in the macaque monkey, though the connections do not overlap but are rather to contiguous, juxtaposed, regions within the IPS (Shipp and Zeki, 1995) (Fig. 8), the connections with V4 lying lateral to those with V5 (see Fig. 6). This segregation in the connections between V4 and V5 on the one hand and parietal cortex on the other is mirrored functionally in human brain by the activation of two contiguous zones within IPS, the activation based on colour grouping lying lateral to the one based on motion grouping, thus arguing for a functional specialization within parietal cortex for groupings based on the two different attributes. Equally, in our study of concept formation, we found that activity in the IPS was segregated in a similar way, depending upon whether subjects recognized stimuli according to colour or motion concepts (Cheadle and Zeki, submitted for publication), thus strengthening our conclusions regarding specialization of function in the IPS. In fact, the specialization within intraparietal sulcus extends beyond grouping for colour and motion. Braddick et al. (2000) have shown that form and motion coherence lead to activity in distinct parts within the intraparietal sulcus. It would be interesting to learn about the foci activated by form coherence relative to those activated by motion and colour. But, collectively, the results speak in favour of a functional specialization for activity related to grouping of different visual attributes within parietal sulcus.

The extensive activation in the IPS that we observed in this study runs antero-posteriorly and hence is orthogonal to the axes of the IPS areas 1–4. The close correspondence between the (MNI) co-ordinates given in the study of Swisher et al. (2007) and the co-ordinates within the band of activation in this one (see Table 2) show that all four subdivisions of the IPS were active during both colour and motion grouping, and that activity in the three anterior ones was parametrically related to the number of groupings. Assuming that these different subdivisions have different functions, it follows that the grouping of visual stimuli according to motion or to colour is of consequence to each. The functions ascribed to posterior parietal cortex include visual attention memory guided saccades (Schluppeck et al., 2006), visual short-term memory (Todd and Marois, 2004) and attention (Silver et al., 2005). How these different functions are distributed across the different subdivisions within IPS is not clear, although both IPS1 and IPS2 appear to be involved in memory guided saccades. All these are functions that could be relevant in our paradigm, but why there should be separate regions devoted to these tasks in relation to colour and motion is both interesting and problematic.

### Grouping and numerosity

Numerosity judgement as a principle involved in grouping has been demonstrated in studies of visual, haptic and auditory perception (Atkinson et al., 1976; Overvliet et al., 2012; ten Hoopen and Vos, 1979). Grouping implicitly involves numbers, since to be grouped there must be one or more elements. If grouping is by exclusion, for example if there is only one solitary element, then this also involves numbers. The parietal cortex, and more specifically the intraparietal sulcus, has also been strongly implicated in numbering and arithmetic abilities (Cohen Kadosh et al., 2008; Park et al., 2012; Pinel et al., 2004). It was therefore interesting to note that the activity in it, for both colour and motion grouping, was parametrically modulated by the number of groupings, the activity being more intense for sets of three groups within each domain than for single groupings or no groupings. Had we used larger groupings we would no doubt have found a more pronounced parametric modulation but the signals that we obtained, even with the modest number of groupings that we used, were so strong as to convince us of a parametric relation between intensity of activity and number of groupings.

Whether the same region of the parietal cortex is engaged with symbolic and non-symbolic numbers has been controversial (see Cohen Kadosh et al., 2008 for a review). What our present results indicate is that numerosity based on colour and that based on motion engage contiguous but distinct parts of the parietal cortex, thus taking the principle of functional specialization beyond the processing of visual signals, to grouping and to numerosity based on different attributes.

### Grouping activity in cerebellum and abstraction

Most papers dealing with numerosity have concentrated on the parietal cortex. In one (Pinel et al., 2004), activity in a region of the cerebellum produced by number/size interference tasks and corresponding to the cerebellar region active in this study was observed but not commented on. The study of Krinzinger et al. (2011) found activity related to symbolic and non-symbolic exact additions in a region of the cerebellum corresponding closely to the region reported here. As well, Bo et al. (2011) have found the same region of the cerebellum as ours to be active in symbolic learning. On the basis of their studies of symbolic representations in the human cerebellum, Balsters and Ramnani (2008) proposed that the posterior, prefrontally-connected part of the cerebellum, and especially lobule HVI of Crus 1 (which showed the largest cerebellar activation in their study and corresponds to the cerebellar region active in this one) is engaged in processing information of an abstract nature. This is a conclusion which the results of this study support, since the parametrically related activity is independent of source, that is to say whether colour or motion groupings. In this context, it is interesting to note that the same region of Crus I was active in a parametrically related fashion for both the colour and motion groupings tasks and, although the activity was more extensive with the colour grouping, it overlapped almost completely with that produced by motion grouping. This overlap is in contrast to the segregation of active areas seen in the parietal cortex, and is itself indicative of its engagement in common – probably symbolic – tasks that may be the hallmark of this area. This is not dissimilar to the activity in the same part of the medial orbito-frontal cortex (field A1) which correlates with the experience of beauty, regardless of whether the source is musical or visual (Ishizu and Zeki, 2011). There, too, the activity is abstract in the sense of not being tied to any source. This leads us to formalize what is obvious, namely that whenever activity in an area resulting from a task derived from, or based on, one source overlaps with activity resulting from a similar task derived from a different source, one must suspect that activity in that area must be related to some kind of abstraction whose basis must be a multi-modal representation.

Whereas most previous studies on attention due to motion have not reported activation in this cerebellar zone, a recent study using attention to motion has linked the cerebellar area active in this study to attentional effects in predictive coding (Kellermann et al., 2012), though it is notable that the frontal cortex (FEF) was also active in their study. We did not observe activity in the cerebellum with motion grouping, which would seem to rule out a general attentional effect, but there was activity there in response to determining the number of motion (or colour) groupings. We would not wish to exclude a possible attentional component in the cerebellar activation in our study, given the mandatory involvement of attention in any grouping and in the determination of the number of groupings. If so, however, the interpretation of our results, using both colour and motion, would imply that this region of the cerebellum is involved in a more abstract way, given the almost total overlap of activity produced by grouping according to colour or to motion.

### Other activations

We obtained strong activity in medial insula in the contrast motion grouping > no grouping but not in the corresponding contrast for colour. The medial insula has been found to be active in a variety of conditions, not necessarily related to visual perception, and its appearance in this contrast and absence in the corresponding colour contrast is puzzling and one for which we have no explanation. This is also true of the activation in the right precentral gyrus shown in the parametric contrast for number of colour groups but not that for motion and for activity in the right inferior frontal gyrus produced by the contrast colour grouping > no grouping.

### Functional specialization beyond V4 and V5

Our main aim in undertaking this experiment was to learn whether the principle of functional specialization extends beyond visual processing, to functions such as grouping which are general in nature. The results show that the principle extends to grouping of colour and motion within parietal cortex, and that this segregation is reflected as well in registering the number of groupings related to the two visual attributes within the IPS. We have shown in the past that the functional specialization in visual cortex for processing different visual attributes is projected in time, entailing a temporal difference in the perception of colour and motion (Moutoussis and Zeki, 1997). Here, we show that this specialization extends beyond the prestriate visual areas, to parietal areas which have a variety of functions related to visual perception (see above). It is of course possible that some kind of integration occurs within parietal cortex itself, through local operations. Even so, our results highlight the importance of the apparently critical strategy of functional specialization and parallel processing not only in processing visual signals but also in perception and in action related to different visual attributes.

## Figures and Tables

**Fig. 4:**
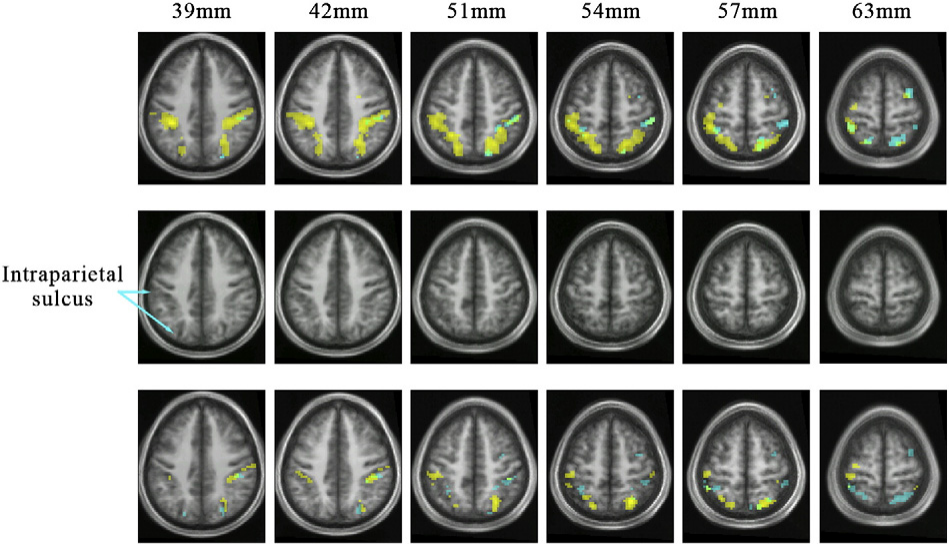
Top row: horizontal sections through the parietal cortex showing activation for the contrasts colour grouping > no grouping (yellow) and motion grouping > no grouping (cyan) within the intraparietal sulcus. (L–R). Middle row: horizontal sections through the parietal cortex to show the location and disposition of the intraparietal sulcus. Bottom row: horizontal sections through the parietal cortex showing activation for the contrasts, for voxels with activity linearly related to the number of colour groups (yellow) and motion groups (cyan) within the intraparietal sulcus. Numbers above each column indicate the MNI level of the horizontal sections.

**Table 1a:** Summary of activations produced by contrasts for colour > greyscale and motion > static. Activations are shown for clusters with p < 0.05 significance after family-wise error correction. P_FWE_ is family-wise error corrected probability for the cluster; K_E_ is cluster size in voxels; X, Y and Z are MNI coordinates in millimetres.

Contrast	Brain areas	P_FWE_ cluster	K_E_	X	Y	Z
Colour > greyscale	rV2, rV4	0.000	742	30	− 70	− 14
Greyscale motion > static	V2, V3, V5 bilaterally	0.000	1718	45	− 61	7
	Parietal	0.032	45	9	− 52	52

**Table 1b:** Summary of activations produced by contrasts for colour groups > no groups, motion groups > no groups, colour no groups > groups and motion no groups > groups. Activations are shown for clusters with p < 0.05 significance after family-wise error correction. Abbreviations as in Table 1a.

Contrast	Brain areas	P_FWE_ cluster	K_E_	X	Y	Z
Colour grouping > no grouping	lV4	0.000	159	− 36	− 55	− 8
	rV4, rV4α and cerebellum	0.000	286	33	− 46	− 23
	Left parietal	0.000	688	− 36	− 37	40
	Right parietal	0.000	712	33	− 43	49
	Right inferior frontal gyrus	0.002	91	57	14	31
Motion grouping > no grouping	lV5	0.000	177	− 36	− 67	2
	rV5	0.000	406	51	− 67	− 2
	Right parietal	0.001	103	27	− 52	70
	Right parietal	0.001	100	48	− 37	58
	Left medial insula	0.000	116	− 39	5	2
	Right medial insula	0.003	51	36	2	4
Colour no grouping > grouping	lV1	0.002	94	− 15	− 97	13
	rV1	0.005	76	18	− 97	10
Motion no grouping > grouping	lV1	0.000	128	− 9	− 97	16

**Table 1c:** Summary of activations produced by contrasts for linear relationships between activation and number of groups. Activations are shown for clusters with p < 0.05 significance after family-wise error correction. Abbreviations as in Table 1a.

Contrast	Brain areas	P_FWE_ cluster	K_E_	X	Y	Z
Parametric by number of colour groups	lV4	0.000	141	− 45	− 70	− 5
	rV4 and cerebellum	0.000	282	33	− 49	− 17
	Left parietal	0.000	116	− 39	− 31	67
	Right parietal	0.001	100	27	− 61	55
	Right parietal	0.047	44	42	− 34	46
	Right precentral gyrus	0.005	72	63	− 16	34
Parametric by number of motion groups	lV5	0.000	309	− 36	− 64	− 2
	rV5	0.000	326	45	− 61	7
	Left parietal	0.006	72	− 36	− 46	58
	Right parietal	0.000	146	27	− 55	67
	Right parietal	0.040	47	30	− 67	28
	Right cerebellum	0.002	87	33	− 46	− 20

**Table 2:** Comparison of IPS area coordinates from Swisher et al., 2007 (top row) with our activations (bottom four rows). The Swisher activations are given as mean positions followed by standard deviations in brackets. A ‘Yes’ indicates that there was activity in our study at the locations given in the top row. Bracketed figures in the bottom four rows indicate the distance from the mean location of the activations in the top row to the nearest suprathreshold voxel in our data. (threshold at p = 0.001 uncorrected).

	IPS1	IPS2	IPS3	IPS4
Swisher	± 23 (6), − 73 (7), 40 (7)	± 21 (5), − 68 (7), 52 (8)	± 25 (7), − 61 (7), 55 (6)	± 26 (6), − 57 (9), 54 (7)
Motion grouping > no grouping	Yes (5 mm)	Yes (5 mm)	Yes (5 mm)	Yes (4 mm)
Colour grouping > no grouping	Yes (0 mm)	Yes (0 mm)	Yes (0 mm)	Yes
Motion groups parametric	Yes (0 mm)	Yes (7 mm)	Yes (4 mm)	Yes (0 mm)
Colour groups parametric	No (7 mm)	Yes (0 mm)	Yes (0 mm)	Yes (0 mm)
