# Supplementary material for: Functional specialization and generalization for grouping of stimuli based on colour and motion
Source: Neuroimage. 2013 Jun;73:156–66. doi: 10.1016/j.neuroimage.2013.02.001 (PMC3613798; doi:10.1016/j.neuroimage.2013.02.001)
Supplement: Supplementary file 1 — Supplementary material. [file mmc1.docx]

Functional Specialisation for Grouping – supplementary

# 1. Retinotopy

The stimulus used for retinotopic mapping was a rotating wedge filled with an expanding, rippling multi-coloured pattern to produce a high-contrast, dynamic colour stimulus defined in non-cartesian coordinates that contained a broad range of spatial frequencies and different orientations. The code to generate the stimulus can be obtained from <http://www.fil.ion.ucl.ac.uk/~sschwarz/retinotopy.html>. The wedge was shown against a mid-grey background. A black fixation cross was drawn at the centre of the screen, and the coloured wedge rotated about the fixation cross. At random intervals a grey circle was drawn on top of the wedge at a random distance from the centre of rotation. Subjects were instructed to focus on the cross and to press a key if they noticed the presence of the grey circle. Ten full rotations of the wedge were shown, each lasting for the duration of twenty scanning volumes (65.28s). Subjects were shown two runs of the stimulus – one clockwise and one anti-clockwise. The stimulus was presented using Psychtoolbox-3 (<http://psychtoolbox.org>) employing the same projection equipment as used for the presentation of stimuli in the main experiment.

Data from retinotopic mapping was pre-processed in SPM8 (http://www.fil.ion.ucl.ac.uk/spm/software/spm8/) to correct for non-linearities in the magnetic field in the scanner and align the functional volumes. The structural image was processed with FreeSurfer (<http://surfer.nmr.mgh.harvard.edu/>) to extract the cortical surface. The functional images were transformed to real and imaginary phase components ([Sereno et al., 1995](#_ENREF_39)) and the clockwise and anticlockwise runs were merged to generate a single average phase map for each subject. The phase map was overlaid on the subject’s inflated cortical surface in FreeSurfer and phase change boundaries were mapped by hand.

# 2. Parametric responses


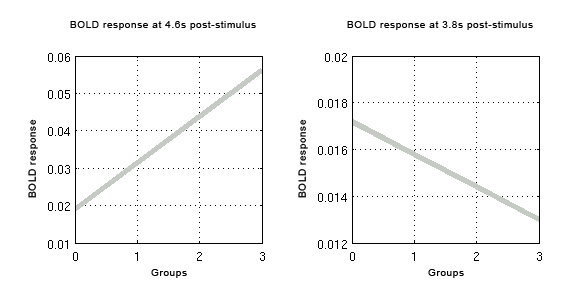


Figure 1 Strongest and weakest relationships between BOLD response and number of colour groups at [-45, -70, -5] MNI (lV4). Post-stimulus times selected according to the peak response in the subjects shown.


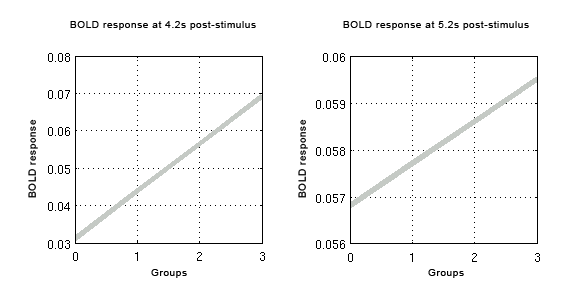


Figure 2 Strongest and weakest relationships between BOLD response and number of motion groups at [45, -61, 7] MNI (rV5). Post-stimulus times selected according to the peak response in the subjects shown.
